# The impact of the scale-up of malaria rapid diagnostic tests on the routine clinical diagnosis procedures for febrile illness: a series of repeated cross-sectional studies in Papua New Guinea

**DOI:** 10.1186/s12936-018-2351-0

**Published:** 2018-05-16

**Authors:** Justin Pulford, Serah Kurumop, Ivo Mueller, Peter M. Siba, Manuel W. Hetzel

**Affiliations:** 1 Papua New Guinea Institute of Medical Research, Goroka, Papua New Guinea; 2 Liverpool School of Tropical Medicine, Liverpool, UK; 3 Walter and Eliza Hall Institute of Medical Research, Melbourne, Australia; 4 Barcelona Centre for International Health Research, Barcelona, Spain; 5 Swiss Tropical and Public Health Institute, Basel, Switzerland; 6 University of Basel, Basel, Switzerland

**Keywords:** Malaria, Diagnosis, RDT, Febrile illness, PNG

## Abstract

**Background:**

This paper examines the impact of the scale-up of malaria rapid diagnostic tests (RDT) on routine clinical diagnosis procedures for febrile illness in primary healthcare settings in Papua New Guinea.

**Methods:**

Repeat, cross-sectional surveys in randomly selected primary healthcare services were conducted. Surveys included passive observation of consecutive febrile case management cases and were completed immediately prior to RDT scale-up (2011) and at 12- (2012) and 60-months (2016) post scale-up. The frequency with which specified diagnostic questions and procedures were observed to occur, with corresponding 95% CIs, was calculated for febrile patients prescribed anti-malarials pre- and post-RDT scale-up and between febrile patients who tested either negative or positive for malaria infection by RDT (post scale-up only).

**Results:**

A total of 1809 observations from 120 health facilities were completed across the three survey periods of which 915 (51%) were prescribed an anti-malarial. The mean number of diagnostic questions and procedures asked or performed, leading to anti-malarial prescription, remained consistent pre- and post-RDT scale-up (range 7.4–7.7). However, alterations in diagnostic content were evident with the RDT replacing body temperature as the primary diagnostic procedure performed (observed in 5.3 and 84.4% of cases, respectively, in 2011 vs. 77.9 and 58.2% of cases in 2016). Verbal questioning, especially experience of fever, cough and duration of symptoms, remained the most common feature of a diagnostic examination leading to anti-malarial prescription irrespective of RDT use (observed in 96.1, 86.8 and 84.8% of cases, respectively, in 2011 vs. 97.5, 76.6 and 85.7% of cases in 2016). Diagnostic content did not vary substantially by RDT result.

**Conclusions:**

Rapid diagnostic tests scale-up has led to a reduction in body temperature measurement. Investigations are very limited when malaria infection is ruled out as a cause of febrile illness by RDT.

## Background

Health worker access to malaria rapid diagnostic tests (RDTs) has increased exponentially over the past decade. Annual global sales increased from approximately 120 to 300 million between 2011 and 2014 [1] and distribution through national malaria control programmes increased fourfold over a similar period [2]. Supported by investment from international development agencies and philanthropic organizations [2], the global scale-up of RDTs has been driven by the widespread adoption of the 2010 WHO recommendation to test all suspected malaria cases prior to administering anti-malarial treatment [3]. The ‘test and treat’ approach represents a step change in febrile case management in malaria endemic countries where laboratory services to support clinical practice are not routinely available. In contrast with former treatment models in which anti-malarial medications were routinely prescribed to febrile patients based on a ‘presumptive’ diagnosis [4], the test and treat protocol stipulates that anti-malarial medication should only be prescribed to patients following parasitological confirmation of malaria infection by microscopy or RDT [5]. This paradigm shift was driven by the changing epidemiology of malaria (particularly decreasing transmission and increasing heterogeneity), the widespread roll-out of more expensive anti-malarial medicines requiring an improved specificity of diagnosis [3] and the increasing availability and affordability of sufficiently sensitive and specific quality-controlled RDTs [6].

Health worker compliance with test and treat protocols, inclusive of RDT use, has been widely investigated [7–10]; however, few studies have examined the potential impact of RDT scale-up on other aspects of diagnostic practice. For example, it is not well understood how better RDT access may have altered the depth and content of a clinical consultation leading to anti-malarial prescription or the way a clinical consultation for febrile illness may vary depending on the result of a RDT. These are important considerations as RDTs are designed to enhance (rather than replace) a comprehensive clinical consultation [5, 11], yet they have been most widely distributed in settings where: clinical standards are frequently poor [12]; health workers have little acquired experience in the clinical diagnosis and treatment of non-malarial febrile patients [13]; and where diagnostic tools to assist in the identification of fever aetiology in RDT negative patients are scarce [14].

This paper examines the impact of RDT scale-up on febrile case management in a low- and middle-income country (LMIC) setting. The research questions include: (1) In what ways does the scale-up of RDTs alter the content of a diagnostic examination leading to anti-malarial prescription, as measured by the number and type of diagnostic questions asked and/or procedures performed? And (2) In what ways does diagnostic examination vary by RDT result, as measured by the number and type of diagnostic questions asked and/or procedures performed?

## Methods

Data were obtained from repeat, countrywide cross-sectional surveys of randomly selected primary healthcare facilities conducted as part of a long-term evaluation of the Papua New Guinea (PNG) National Malaria Control Programme. A full description of the evaluation programme, including a detailed description of the health facility survey methodology, is presented elsewhere [15].

### Study setting

Papua New Guinea is a country of approximately 7.3 million people situated in the South West Pacific. Malaria endemicity ranges from endemic transmission in lowland and coastal settings to unstable transmission with localized epidemics in many Highlands areas [16]. Over 90% of the population are considered at risk of malaria infection [2], although estimated national malaria prevalence has fallen to a historic low of 0.9% following substantial investment in the National Malaria Control Programme [17]. Most primary healthcare services in PNG are delivered through a network of government- and church-provided health centres, health sub-centres and aid posts in adherence with National Health Service Standards [18]. In principle, all cases of uncomplicated malaria should be treatable at primary healthcare facilities which are almost exclusively staffed by nurses and community health workers [19]. PNG implemented ‘test and treat’ malaria case management guidelines in late 2011, replacing the presumptive treatment guidelines previously in place [20]. The percentage of health centres with RDTs in stock subsequently increased from 17.5% in 2010 [19] to 90.2% in 2012 [21].

### Study sample

A stratified approach was used to select health facilities, with two health centres or health sub-centres (collectively referred to as health centres in this paper) selected from each province using a simple random sampling procedure. The sampling frame included all health centres operational in March 2010 inclusive of government and mission administered health facilities (N = 689). Sample selection was conducted anew for each survey. All febrile patients meeting eligibility criteria attending selected health facilities during survey periods were recruited consecutively. Patients were considered eligible for participation if they were outpatients presenting with febrile symptoms, reported a recent history of fever and had not been treated for malaria infection in the past 14 days (to exclude ‘treatment review’ cases). Eligible patients were identified upon first contact with a health worker or, if circumstances allowed, by screening in the waiting area prior to first contact with a health worker.

### Survey procedure

The three surveys were carried out from June to November in 2011 and 2012 and from February to July in 2016. The 2011 survey was completed in the period immediately prior to the introduction of the test and treat guidelines, the 2012 and 2016 surveys were completed post-implementation. Survey data were collected by trained field teams working simultaneously at different sites. Members of each survey team spent 3–5 days at each participating health centre collecting a range of data. This paper reports data obtained from non-participant observations of fever or suspected malaria case management.

### Survey instrument

The non-participant observation was based on a structured checklist designed to record observed features of the clinical case management of patients presenting with fever or a recent history of fever. The instrument was divided into discrete sections including consultation and diagnosis, prescription and treatment counselling. The content of each section was informed with input from experienced medical- and medical research-professionals. The instrument was completed by a trained field team member who would silently observe the management of patients from the point of initial contact with a health professional until service exit or admission onto a treatment ward. During the course of this observation, the field team member would record whether specified actions did or did not occur as well as the outcome of specific actions (e.g. whether an RDT was conducted and, if yes, the RDT result).

The data presented in this study are largely drawn from the ‘diagnosis’ section of this checklist. For the 2011 and 2012 surveys, this section solely comprised 16 questions or procedures (listed in Table 2) that were considered essential to a thorough diagnostic examination of suspected malaria. The greater the number of questions/procedures completed, the more comprehensive the diagnostic examination (and vice versa). For the 2016 survey, an additional eight questions or procedures were added to this section (listed in Table 5) to account for differential diagnosis in the event of a confirmed non-malaria febrile illness (NMFI), i.e. additional/alternative questions/procedures that a health worker might be expected to complete once a malaria infection has been ruled out by RDT. A ‘question’ was considered to have been completed irrespective of whether an answer was obtained via direct health worker enquiry or via unprompted patient disclosure.

### Data analysis

All data were double entered into DMSys version 5.1 (Sigma Soft International). Stata/SE version 12 was used for descriptive data analysis and for calculating 95% confidence intervals (CIs). The calculation of all CIs was adjusted for possible clustering at the health facility level using the Stata ‘svy’ command set in which health facilities were defined as the primary sampling unit. Between group differences in observed diagnostic practice (by survey year or by RDT result), inclusive of the frequency with which each diagnostic question/procedure was completed and the mean number of questions/procedures completed, were examined by Chi square, t test or one-way ANOVA as appropriate. The rank order of diagnostic questions/procedures (from most- to least-common) is also presented to highlight changes over time in diagnostic examination leading to anti-malarial prescription, or between group differences in diagnostic examination based on RDT result.

Febrile patients who were admitted or referred elsewhere for additional treatment were excluded from analysis (i.e. severely ill patients). Analyses examining research question two (In what ways does diagnostic examination vary by RDT result?) were limited to 2016 data as this was the only year in which additional diagnostic questions that may be asked of confirmed NMFI patients were included.

## Results

### Diagnostic practice leading to anti-malarial prescription

A total of 1809 observations from 120 health facilities were completed across the three survey periods of which 915 (51%) were prescribed an anti-malarial. Anti-malarial prescriptions were provided to 506/582 (87%) patients from 43/44 health facilities in 2011, 165/426 (39%) patients from 28/37 health facilities in 2012 and 244/801 (30%) patients from 30/39 health facilities in 2016. Selected characteristics of the febrile patients prescribed anti-malarials in the three survey samples are presented in Table 1. Between group variation in age was statistically significant (p < 0.005), with fewer participants in the < 5 year age band in 2012 and 2016 as compared to 2011.

**Table 1:** Selected participant characteristics by survey year

Characteristic	2011	2012	2016
	(N = 506)	(N = 165)	(N = 244)
	n (%)	n (%)	n (%)
Sex			
Male	242 (48)	79 (48)	130 (53)
Female	264 (52)	86 (52)	114 (47)
Age (years)			
< 5	273 (55)	63 (38)	77 (32)
5–14	224 (45)	102 (62)	166 (68)

Table 2 presents the percentage (95% CI) of febrile patients prescribed anti-malarials who were observed being asked each of nine specified diagnostic questions or who were observed receiving each of seven diagnostic procedures. A decrease in frequency was observed between 2011 and 2016 on 10/16 questions/procedures; seven of which were statistically significant. An increase in frequency was observed for the remaining six questions/procedures across the same time-period of which five were statistically significant. The greatest observed decrease was in the percentage of febrile patients who had their temperature taken (84–58%). The greatest observed increase was in the percentage of febrile patients receiving a RDT or blood slide (5–78%).

**Table 2:** Frequency of diagnostic questions and procedures leading to anti-malarial prescription by survey year

Questions asked and procedures performed	2011 (N = 506)	2012 (N = 165)	2016 (N = 244)	*p*	Rank order		
	% (95% CI)	% (95% CI)	% (95% CI)		2011	2012	2016
Questions							
Current use of any medication	26.1 (17.9, 36.4)	27.9 (19.4, 38.3)	24.2 (16.4, 34.2)	0.698	10	11	12
Concurrent illness/existing condition	48.6 (38.8, 58.5)	44.9 (35.7, 54.3)	34.0 (25.1, 44.2)	< 0.005	9	10	10
Experience of fever	96.1 (92.8, 97.9)	89.7 (74.3, 96.3)	97.5 (94.4, 99.0)	< 0.005	*1*	*1*	*1*
Experience of cough	86.8 (82.7, 90.0)	80.0 (69.3, 87.7)	76.6 (64.3, 85.7)	< 0.005	*2*	*3*	*4*
Experience of head/body ache/pain	58.5 (51.7, 65.0)	56.4 (41.0, 70.6)	64.3 (53.3, 74.1)	0.197	8	9	*5*
Experience of nausea/vomiting	65.6 (59.5, 71.3)	63.0 (51.5, 73.2)	61.5 (51.3, 70.8)	0.519	6	6	6
Experience of diarrhoea	65.4 (57.9, 72.3)	58.8 (46.7, 69.9)	57.8 (46.1, 68.7)	0.080	7	8	9
Experience of chills	20.0 (13.9, 27.8)	27.3 (19.4, 36.9)	32.8 (22.9, 33.9)	< 0.005	11	12	11
Duration of current symptoms	84.8 (79.8, 88.7)	77.0 (61.6, 87.5)	85.7 (76.2, 91.8)	0.038	*3*	*4*	*2*
Procedure							
Body temperature measured	84.4 (75.9, 90.3)	81.8 (63.3, 92.2)	58.2 (40.1, 74.3)	< 0.005	*4*	*2*	8
Body weight measured	77.7 (69.2, 84.3)	73.9 (58.1, 85.3)	60.3 (45.7, 73.2)	< 0.005	*5*	*5*	7
Blood pressure measured	0.6 (0.2, 1.8)	6.1 (2.6, 13.3)	0.8 (0.2, 3.4)	< 0.005	16	15	15
Abdomen palpated	14.0 (10.1, 19.1)	12.1 (7.0, 20.1)	6.6 (2.5, 15.9)	0.011	13	13	13
Eyes examined	15.2 (10.4, 21.7)	7.9 (4.2, 14.2)	3.7 (1.1, 12.1)	< 0.005	12	14	14
Palms examined	1.6 (0.5, 4.5)	3.6 (1.4, 9.3)	0 (–)	0.013	15	16	16
Malaria RDT/blood slide completed	5.3 (3.0, 9.4)	60.6 (34.4, 81.9)	77.9 (58.0, 90.0)	< 0.005	14	7	*3*

Five most frequently observed questions or procedures are in italics

Table 3 presents the respective availability of the resources necessary to complete the diagnostic procedures listed in Table 2 in participating health facilities per survey year. The availability of resources varied by type, although were generally consistent over time. Variation in RDT availability was statistically significant (p < 0.005); variations in the availability of other resources were not.

**Table 3:** Number and percent of health facilities with specified resources available

Characteristic	2011	2012	2016
	(N = 44)	(N = 37)	(N = 39)
	n (%)	n (%)	n (%)
Thermometer	42 (95)	37 (100)	38 (97)
Bodyweight scale	44 (100)	35 (95)	37 (95)
Blood pressure apparatus	38 (86)	26 (70)	32 (82)
RDTs	10 (23)	33 (89)	28 (70)
Microscopy	5 (11)	1 (3)	3 (8)
Anti-malarials	44 (100)	37 (100)	39 (100)

Health facilities from which one or more of the clinical observations took place

The rank order, in terms of most to least observed, of the specified diagnostic questions and procedures by survey year is presented in Table 2. The five most frequently observed questions or procedures were consistent in 2011 and 2012, although two changes were evident by 2016. These included the use of RDT or microscopy to diagnose malaria infection (ranked 3rd in 2016, up from 7th in 2012 and 14th in 2011) and experience of head/body pain (ranked 5th in 2016, up from 9th in 2012 and 8th in 2011) at the expense of measuring body weight and temperature (ranked 7th and 8th, respectively, in 2016).

**Fig. 1:**
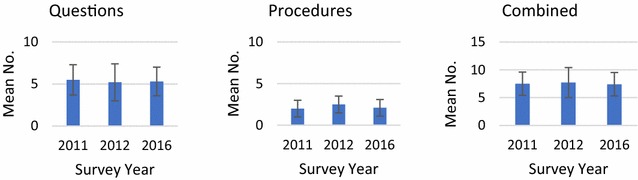
Mean number (standard deviation) of diagnostic questions, procedures and combined questions/procedures observed by survey year

Minor variations in the mean number of questions (5.5, 5.2, 5.3), procedures (2.0, 2.5, 2.1) and combined questions/procedures (7.5, 7.7, 7.4) asked or performed were evident between 2011, 2012 and 2016 (Fig. 1), although only the variation in procedures was statistically significant (*p *< 0.005).

### Differential diagnosis

Rapid diagnostic tests status was available for 781/801 (98%) of participants from all 39 health facilities included in the 2016 survey, of whom 210/781 (27%) did not receive an RDT, 405/781 (52%) received an RDT and tested negative for malaria infection and 166/781 (21%) received an RDT and tested positive for malaria infection. Selected demographic and prescription data for the three diagnostic groups are presented in Table 4. Between group variations in sex ratio were not statistically significant, but between group variations in age and in the proportions prescribed anti-malarials, antibiotics and analgesics were (all p < 0.005). The variations in the proportions prescribed anti-malarials, antibiotics and analgesics remained statistically significant when just comparing the RDT negative versus RDT positive sub-groups (all p < 0.005), although sex and age differences were not.

**Table 4:** Participant sex, age and prescription by RDT result, 2016 sample only

Characteristic	Not tested	RDT−	RDT+
	(N = 210)	(N = 405)	(N = 166)
	n (%)	n (%)	n (%)
Sex			
Male	111 (53)	200 (49)	73 (44)
Female	99 (47)	205 (51)	93 (56)
Age (years)			
< 5	104 (50)	160 (40)	52 (32)
5+	196 (50)	245 (60)	114 (68)
Prescription^a^			
Anti-malarial	54 (26)	23 (6)	166 (100)
Antibiotic	160 (76)	337 (83)	43 (26)
Analgesic	125 (60)	305 (75)	98 (59)

^a^ Patients may have been prescribed more than one medication

Table 5 compares those febrile patients who tested positive for malaria infection by RDT with those who tested negative on a broader range of diagnostic questions and procedures. Figures and percentages for those febrile patients who were not provided a RDT are also presented to afford a further comparison, although this sub-group was excluded from further analysis. Overall, 12/23 questions/procedures were more frequently observed among RDT positive patients as compared to RDT negative patients, of which three were statistically significant. Eleven questions/procedures were more frequently observed among patients who tested negative for malaria infection, three of which were statistically significant. The greatest observed difference between the RDT negative and positive patient groups was in the percentage asked whether they had a concurrent illness or existing condition (18.9% in RDT− patients vs. 33.7% in RDT+).

**Table 5:** Frequency of diagnostic questions and procedures by RDT result, 2016 sample only

Questions asked and procedures performed	Not tested (N = 210)	RDT− (N = 405)	RDT+ (N = 166)	*p* ^a^	Rank order	
	% (95% CI)	% (95% CI)	% (95% CI)		RDT−	RDT+
Questions						
Current use of any medication	23.3 (14.8, 34.8)	16.3 (9.4, 26.9)	24.1 (15.2, 36.0)	0.029	12	11
Concurrent illness/existing conditions	35.2 (24.8, 47.3)	18.9 (14.1, 24.9)	33.7 (23.5, 45.8)	< 0.005	11	9
Experience of convulsions	3.8 (1.3, 11.1)	2.6 (1.1, 6.0)	3.6 (1.7, 7.7)	0.515	19	19
Experience of drowsiness/unconsciousness	7.7 (4.3, 13.3)	9.7 (5.9, 15.6)	8.4 (4.2, 16.3)	0.631	15	15
Experience of fever	98.6 (95.9, 99.5)	97.9 (96.1, 98.9)	97.6 (94.4, 99.0)	0.834	*1*	*1*
Experience of cough	87.1 (70.9, 95.0)	84.4 (77.7, 89.3)	78.3 (68.0, 86.0)	0.079	*3*	*3*
Experience of breathing difficulties	40.0 (24.8, 57.4)	31.5 (22.2, 42.6)	21.1 (12.1, 34.2)	0.012	9	12
Experience of head/body pain	53.3 (44.1, 62.3)	57.9 (50.0, 65.4)	65.1 (51.7, 76.4)	0.112	*5*	*4*
Experience of ear ache	14.8 (7.9, 26.2)	10.9 (6.6, 17.6)	11.5 (6.4, 19.8)	0.857	14	13
Experience of nausea/vomiting	59.1 (45.3, 71.5)	54.4 (45.6, 62.9)	59.6 (40.1, 68.5)	0.247	7	7=
Experience of diarrhoea	61.4 (46.7, 74.3)	55.3 (46.7, 63.7)	60.2 (48.5, 70.9)	0.278	6	6
Experience of chills	32.4 (21.2, 46.1)	23.2 (17.7, 29.7)	28.3 (19.5, 39.2)	0.192	10	10
Ability to drink/breastfeed	30.0 (19.0, 45.0)	15.4 (10.1, 22.8)	9.0 (4.7, 16.6)	0.043	13	14
Measles/spots in past 3 months	4.3 (2.4, 7.6)	2.4 (0.9, 6.3)	0 (–)	0.045	20=	22=
Duration of current symptoms	89.5 (81.9, 94.2)	85.3 (76.3, 91.4)	84.3 (73.6, 91.2)	0.758	*2*	*2*
Procedure						
Body temperature measured	59.1 (41.2, 74.8)	67.9 (49.2, 82.1)	59.6 (39.7, 76.9)	0.059	*4*	7=
Body weight measured	45.2 (30.9, 60.5)	53.0 (41.5, 64.1)	63.9 (45.1, 79.1)	0.016	8	*5*
Blood pressure measured	3.8 (0.8, 16.7)	0.2 (0.1, 1.9)	1.2 (0.3, 5.0)	0.137	23	20=
Abdomen palpated	6.7 (3.1, 13.8)	3.6 (1.6, 7.5)	7.2 (2.1, 22.3)	0.055	18	17
Eyes examined	11.0 (5.4, 21.1)	6.4 (3.3, 11.9)	4.2 (0.9, 18.2)	0.311	16	18
Palms examined	1.9 (0.4, 9.0)	1.9 (0.8, 4.7)	0 (–)	0.074	22	22=
Respiratory rate measured	16.7 (7.1, 34.2)	4.3 (2.2, 8.2)	7.9 (2.5, 22.8)	0.074	17	16
Other diagnostic test (or referral)	1.4 (0.3, 6.1)	2.4 (0.9, 6.3)	1.2 (0.2, 7.5)	0.194	20=	20=

Five most frequently observed questions or procedures are in italics

^a^ Based on Chi square analysis of ‘RDT−’ vs. ‘RDT+’ sub-groups only (i.e. data presented in the ‘not tested’ column were excluded from this test)

The rank order, in terms of most to least observed, of the specified diagnostic questions and procedures by RDT result is presented in Table 5. Four out of the five most frequently observed questions or procedures were consistent across both RDT negative and RDT positive patient groups, including: experience of fever, experience of cough, experience of head or body pain and duration of current symptoms.

**Fig. 2:**
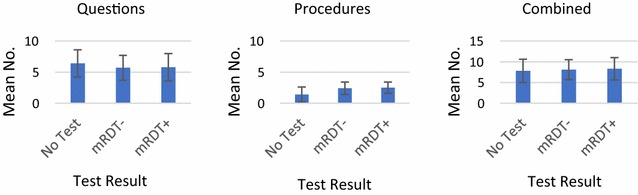
Mean number (standard deviation) of diagnostic questions, procedures and combined questions/procedures observed by RDT result (2016 only)

No statistically significant differences in the mean number of questions (5.7 vs. 5.8), procedures (2.4 vs. 2.5) or combined questions/procedures (8.1 vs. 8.3) asked or performed were observed between RDT− and RDT+ groups (Fig. 2). The mean number of questions asked was greater for febrile patients not tested for malaria infection (6.4), although the mean number of procedures completed was lower (1.4) as was the overall number of combined questions/procedures (7.8).

## Discussion

This study found that the mean number of diagnostic questions and procedures asked or performed, leading to anti-malarial prescription, remained consistent pre- and post-RDT scale-up. Thus, the widespread introduction of an additional diagnostic procedure (i.e. the RDT) did not increase the content of diagnostic examination. Rather, alterations in diagnostic content were evident with the RDT replacing body temperature as the most commonly employed diagnostic procedure (the former increasing in frequency from 5.3 to 77.9%, the latter decreasing from 84.6 to 58.2%). Measuring bodyweight also became less common across the three survey periods, although a greater proportion of participants were aged over 5 years in the latter two surveys (and were, therefore, perhaps less likely to be weighed) which may have accounted for this result. Verbal questioning, especially experience of fever, cough and duration of symptoms, remained the most common feature of a diagnostic examination leading to anti-malarial prescription irrespective of RDT use. Content-related changes to diagnostic examination following RDT scale-up, therefore, appear to be relatively minimal and primarily limited to a reduction in the frequency of body temperature measurement.

Similarly, the study found that diagnostic content did not vary substantially by RDT result (RDT− vs. RDT+). The mean number of diagnostic questions and procedures asked or performed were markedly similar irrespective of test result and there were only minor between-group variations in the frequency with which each of 23 specified diagnostic questions or procedures were observed. In addition, febrile patients not tested for malaria infection by RDT in the 2016 survey were no more likely to have their body temperature measured as compared to those who were tested by RDT, suggesting a ‘culture shift’ away from routine body temperature measurement in cases of febrile illness may have taken place.

The study findings do not challenge the benefit of RDT scale-up to febrile patients prescribed anti-malarials based on a confirmed malaria infection. The WHO approved RDTs widely used in PNG have sound metrics [22], RDT-based malaria case management has been proven effective in PNG [23] and national survey data indicate artemisinin combination therapies (ACTs) are widely prescribed to confirmed malaria cases [21]. Whether body temperature is measured or not, a positive RDT result, if followed up by an appropriate, ACT-based anti-malarial prescription, is likely to result in a more cost-efficient and effective treatment than that provided under the former presumptive model of malaria case management.

Benefits of RDT scale-up in NMFI cases are less clear. Febrile patients who test negative for malaria infection by RDT are less likely to receive an unnecessary anti-malarial medication as compared to the near universal prescription of anti-malarials under the former presumptive approach [24]. However, many NMFI patients are not receiving a more intensive or varied diagnostic examination (as may be expected following ‘the ruling out’ of malaria infection by RDT) and the rate of antibiotic prescription (83%) is alarmingly high. A recent study of health worker adherence to national prescription guidelines in PNG found antibiotics were overprescribed in 41% of over 3000 NMFI cases across 10 specified illnesses [25] and international evidence suggests antibiotic over prescription in NMFI is a growing concern [26]. Ensuring appropriate management of NMFI is increasingly important in PNG as malaria case positivity rates have dropped (often well) below fifty percent [27] in response to an effective national malaria control programme [17].

Further inadequacies in primary healthcare workers’ diagnostic practice were evident pre- and post RDT scale-up. Contextually appropriate and accessible diagnostic procedures such as abdomen palpation were rarely conducted nor were common ‘danger signs’ routinely investigated. A statistically significant reduction in the frequency with which concurrent illnesses or existing conditions were queried among patients prescribed anti-malarials was also observed across time, suggesting the confirmation of malaria by RDT may discourage health workers from exploring other/existing causes of illness. When one also considers the limited laboratory support for the diagnosis of NMFI in PNG and poor treatment counselling practices previously documented [21, 24], then it is difficult not to conclude that the level of training, support and supervision provided to primary healthcare workers to support accurate diagnosis and treatment of febrile illness is insufficient.

The reported study was not without limitation. Participating clinicians were aware that they were being observed and may have altered their clinical practice accordingly. The expected effect of any such bias would be towards perceived ‘better’ practice. The analysis of diagnostic practice leading to anti-malarial prescription was limited to diagnostic features considered important or essential to a malaria diagnosis and did not account for other diagnostic questions or procedures that may have been asked or completed. The sequence with which specified diagnostic questions/procedures were completed was not recorded. However, in practice, all/most questions were typically asked prior to completing a RDT or obtaining the RDT result. Finally, the sample did not include any observations from the lowest level of primary healthcare service provision in PNG, the aid-post (typically staffed by a single health worker in remote locations), and so the reported findings should not be considered reflective of diagnostic practice at this level.

The study findings and implications presented in this paper are unlikely to be unique to PNG. Challenges related to health worker practice, training and supervision in resource poor settings [12] are common to many malaria endemic countries. Intensive investment in vertical disease control programmes, such as occurred in PNG, can potentially undermine health systems strengthening [28, 29]. The few studies that have examined the impact of investment in malaria control programmes on the management of febrile illness at a broader level (i.e. analyses not restricted to confirmed malaria cases only) have identified reduced or sub-optimal systems effectiveness, despite improvements in malaria case management [26, 30, 31]. Findings from this study do not suggest febrile case management has worsened in PNG as a result of RDT scale-up; however, they do suggest that the implications of RDT scale-up on wider febrile case management were not as well considered as they could have been. Arguably, the dedicated training resources provided in support of RDT scale-up in PNG, such as multi-day ‘skills’ workshops for healthcare workers and the development and dissemination of programme specific job-aides [32], presented an opportunity to strengthen diagnostic, prescription and counselling practices across a board array of febrile illnesses. However, the training focus was restricted to malaria case management and, as such, the opportunity for broader health systems strengthening was lost.

## Conclusions

Primary healthcare workers in PNG are using RDTs in their diagnostic examination of febrile illnesses at a substantially higher rate following nationwide scale-up, although the level of assessment remains similar as complementary diagnostic procedures are used less often. In those cases where malaria infection has been ruled out as a cause of febrile illness by RDT, few additional or alternative questions or procedures are undertaken beyond those routinely completed anyway. The standard of diagnostic examination remains sub-optimal irrespective of the cause of febrile illness. Health worker performance may be enhanced by providing training or support initiatives that pertain more broadly to febrile case management as opposed to malaria-specific case management.
